# Value of the spot sign combined with the blend sign for predicting the outcome of patients with intracerebral hemorrhage who have undergone stereotactic minimally invasive surgery

**DOI:** 10.3389/fneur.2025.1577675

**Published:** 2025-08-06

**Authors:** Wei Che, Lijuan Zhu, Likun Wang, Qian Wu, Lei Huang, Fei Ye, Siying Ren, Guofeng Wu

**Affiliations:** ^1^Emergency Department, The Affiliated Hospital of Guizhou Medical University, Guiyang, China; ^2^Department of Neurology, The Affiliated Hospital of Guizhou Medical University, Guiyang, China

**Keywords:** spot sign, minimally invasive surgery, intracerebral hemorrhage, blend sign, spot sign combined with the blend sign

## Abstract

**Objective:**

This study aimed to explore the effect of combining the computed tomographic angiography (CTA) spot sign with the computed tomography (CT) blend sign for predicting the outcome of patients with intracerebral hemorrhage (ICH) who have undergone stereotactic minimally invasive surgery (sMIS).

**Methods:**

Patients with ICH treated with sMIS between January 2018 and April 2023 at the affiliated hospital of Guizhou Medical University were retrospectively included. A modified Rankin scale (mRS) score of ≥3 at 3 months was defined as a poor outcome. According to neurological recovery, patients were assigned to either the poor outcome (90 patients) or the good outcome (105 patients) groups. The value of the combined signs in predicting the outcome of patients with ICH who have undergone sMIS was analyzed via the receiver operating characteristic (ROC) curve.

**Results:**

Among a total of 195 patients, 29 (14.8%) had single blend signs, 35 (17.9%) had single spot signs, 76 (39%) had combined signs, and 55 (28.2%) had CT-negative. A multivariate binary logistic regression analysis revealed that the spot sign combined with the blend sign (OR = 5.244, 95% CI: 2.606–10.55, *p* < 0.001) and hematoma expansion (HE) (OR = 2.063, 95% CI: 1.003–4.245, *p* = 0.049) were associated with poor outcomes (*p* < 0.001). The ROC curve analysis revealed that the sensitivity and specificity of the spot sign and blend sign combination for outcome prediction were 61.1 and 80.0%, respectively; the Youden index was 0.411, and the area under the curve (AUC) value was 0.706.

**Conclusion:**

The spot sign combined with the blend sign and hematoma topography may have predictive value for outcomes in patients with ICH undergoing sMIS.

## 1 Introduction

Intracerebral hemorrhage (ICH) is a fatal type of stroke, with a 1-month mortality rate of approximately 40% and severe neurological deficits affecting approximately 75% of surviving patients (1, 2). Early and extensive hematoma evacuation is a crucial therapeutic target, typically performed clinically via craniotomy or stereotactic minimally invasive surgery (sMIS). Compared with craniotomy, sMIS has been shown to improve long-term outcomes in hypertensive ICH, especially in cases of deep hemorrhages (3–5). The use of sMIS for ICH has increased significantly and appears to be associated with decreased mortality (6). However, postoperative rebleeding remains a major complication of sMIS, adversely affecting patient prognosis (7). Despite this well-recognized complication, there are relatively few proven prognostic predictors for patients with ICH undergoing sMIS.

Neuroimaging indicators obtained from CT, including the blend (8), black hole (9), and CTA spot (10) signs, can also assist in predicting HE. The CT blend and CTA spot signs have been linked to adverse outcomes in patients with ICH after medication administration (11, 12). In our previous study, we reported that the presence of the CT blend sign is closely associated with postoperative hemorrhage in patients with ICH who have undergone sMIS (13). Moreover, we found that the blend sign demonstrates no correlation with poor outcomes in patients with ICH after sMIS, and patients exhibiting the blend sign should undergo sMIS if they are deemed suitable candidates for the procedure (14). However, these two studies failed to evaluate the predictive value of spot signs for the outcome of patients with ICH who have undergone sMIS. The prognostic value of spot signs combined with blend signs for poor outcomes in sMIS patients remains unconfirmed.

Hence, this study aimed to retrospectively determine the predictive value of the CTA spot sign combined with the CT blend sign for outcomes in patients with ICH following sMIS.

## 2 Methods

### 2.1 Study design and population

Consecutive patients with ICH admitted to the Emergency Department of the Affiliated Hospital of Guizhou Medical University between January 2018 and April 2023 were enrolled in this retrospective study. The ICH diagnosis was confirmed by imaging examination at the time of admission. The study protocol was approved by the Ethics Committee of the Guizhou Medical University Ethical Board (2019/114) and the Affiliated Hospital of Guizhou Medical University (Ethics Approval No. 2022 Ethics No. 91). The patients or their families signed informed consent for treatment.

The inclusion criteria for patients were as follows: (1) those aged ≥18 years and met the indications for surgery, including lobe and basal ganglia hemorrhage ≥30 mL, thalamic and cerebellar hemorrhage ≥10 mL, and brainstem hemorrhage ≥5 mL; (2) those who underwent brain CTA within 6 h of admission, had optimized CT localization before surgery, and had head CT scans reviewed in the first 24 h after the operation; and (3) those who had no contraindications to the operation.

The exclusion criteria were as follows: (1) patients with a long history of oral anticoagulant use or a history of hematologic diseases; (2) patients with secondary ICH due to cerebral vascular malformation, intracranial aneurysm, cerebral tumor, cerebral trauma, or cerebral infarction with hemorrhagic transformation and subcortical hemorrhage due to cerebral amyloid angiopathy; (3) patients with primary ventricular hemorrhage; and (4) patients with incomplete data.

### 2.2 Data collection and definition

General clinical data were collected, including patient sex, hypertension status, age, diabetes mellitus status, smoking history, alcohol consumption history, blood pressure at admission, baseline hematoma volume at admission, Glasgow Coma Scale (GCS) score, and National Institute of Health Stroke Scale (NIHSS) score. Laboratory test results at admission included blood glucose levels, hyperhomocysteinemia, cystatin, serum calcium, and serum sodium levels. Initial CT imaging characteristics recorded comprised hematoma locations (the basal ganglia, lobar regions, brainstem, thalamus, and cerebellum), and imaging features such as the spot sign, blend sign, and their combination. Intracranial pressure was assessed before and after surgery. Furthermore, the number of days of cerebral drain retention and ICU admission, and the presence of postoperative pulmonary infections, gastrointestinal hemorrhage, and heart failure were monitored.

The ICH measurements were performed using the Tada formula method (15), where hematoma volume (ml) = longest diameter (cm) × width (cm) × layer thickness (cm)/2. For irregular hematomas, the volume was calculated using the formula *V* = 2/3SH, where *S* represents the area of the largest cross-section of the hematoma, and *H* is derived by multiplying the number of layers of the hematoma by the thickness of the hematoma cross-section.

**Figure 1:**
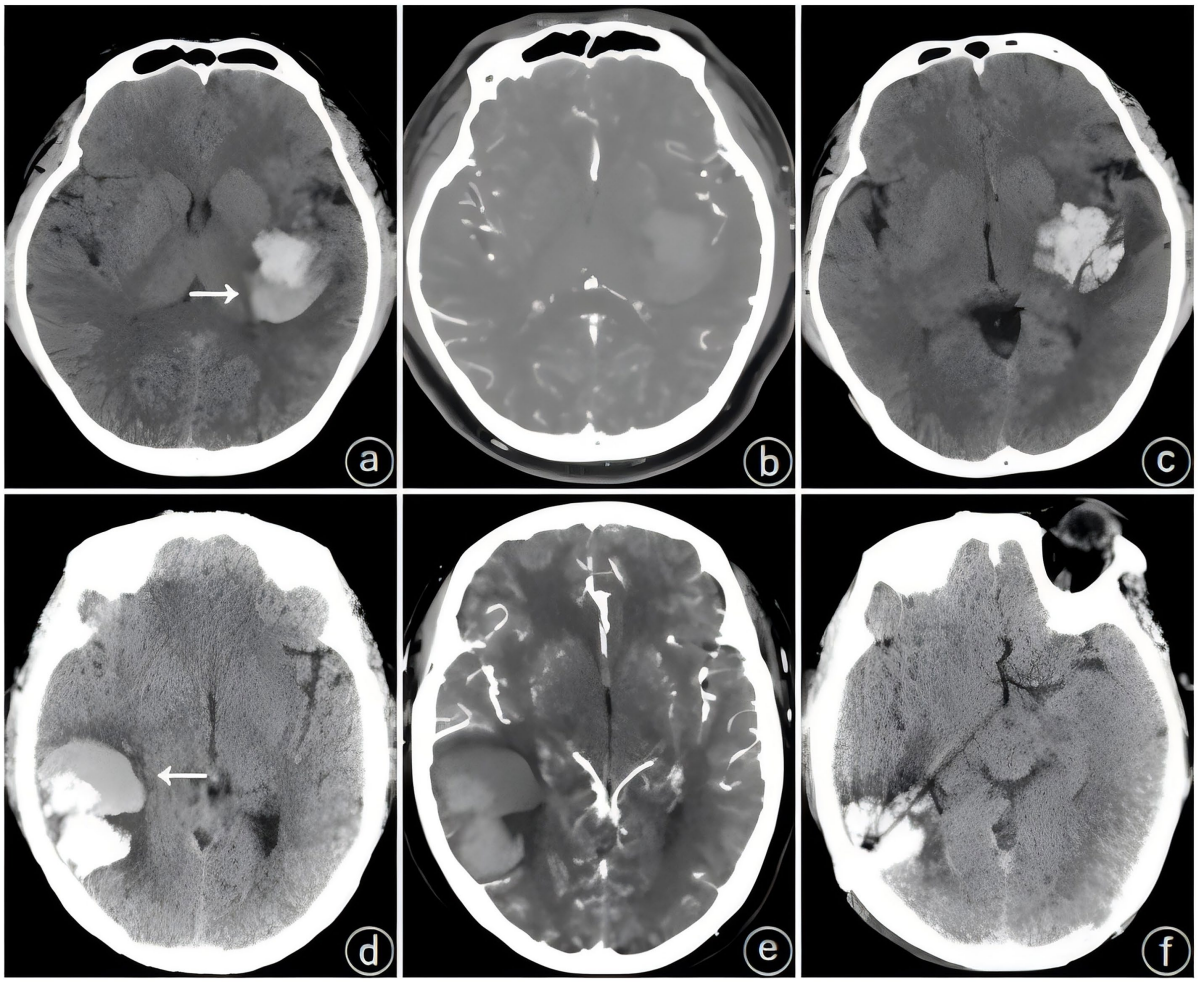
Typical cases of a single blend sign (white arrow). **(a–c)** Patient: male, 35 years old, **(a)** initial hematoma 28 mL, **(b)** no spot sign on computed tomographic angiography (CTA) within 24 h, **(c)** postoperative review of hematoma expansion (HE). **(d–f)** Patient: female, 49 years old, **(d)** initial hematoma 30 mL, **(e)** no spot sign on computed tomographic angiography (CTA) within 24 h, **(f)** postoperative review of no hematoma expansion (HE).

**Figure 2:**
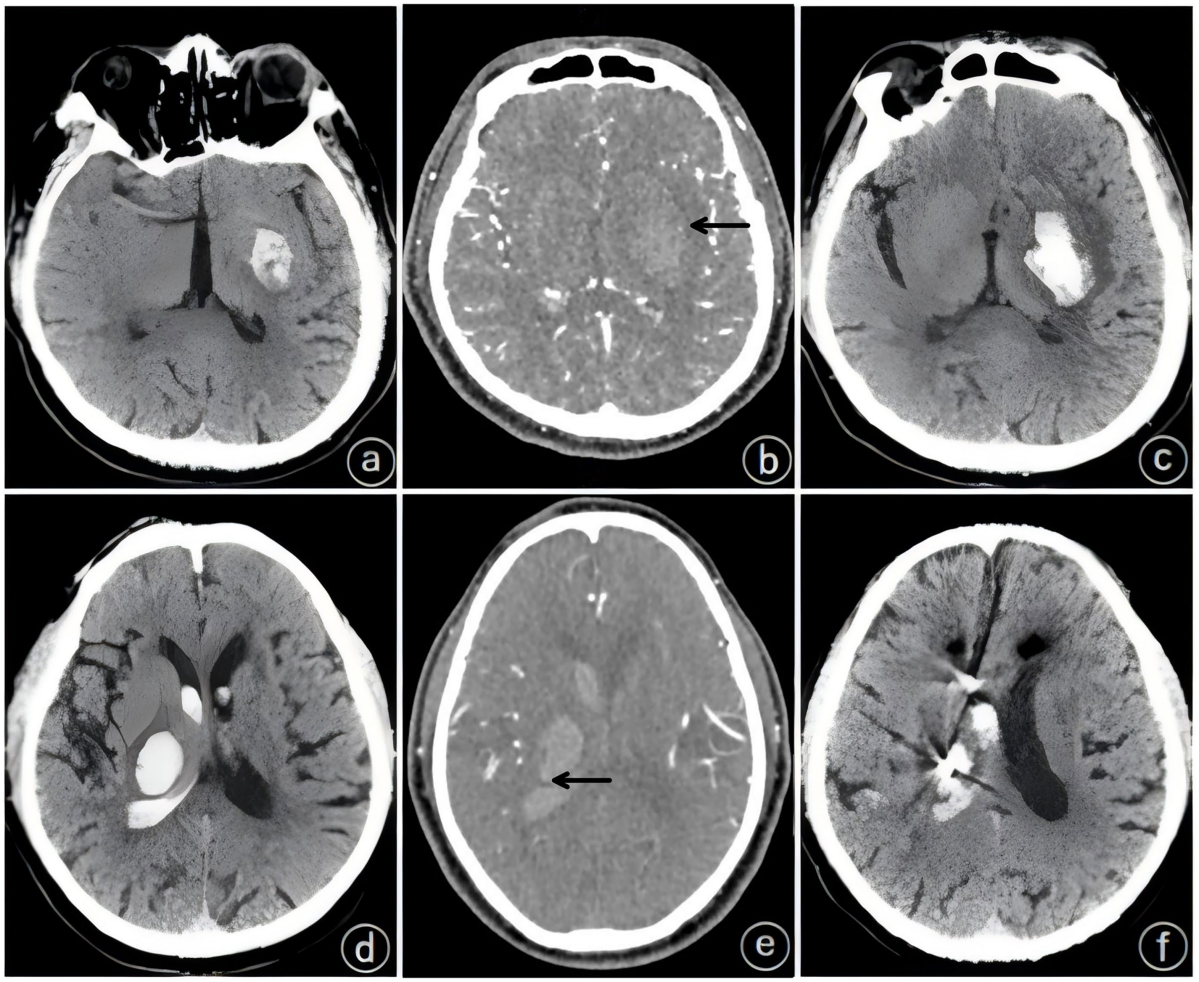
Typical cases of a single spot sign (black arrow). **(a–c)** Patient: male, 58 years old, a: initial hematoma of 15 mL, **(b)** spot sign seen on computed tomographic angiography (CTA) within 24 h, **(c)** hematoma expansion (HE) on preoperative repeat computed tomography (CT). **(d)** Patient: male, 74 years old, initial hematoma of 23 mL; **(e)** spot sign on computed tomographic angiography (CTA) within 24 h; **(f)** no hematoma expansion (HE) on postoperative repeat computed tomography (CT).

**Figure 3:**
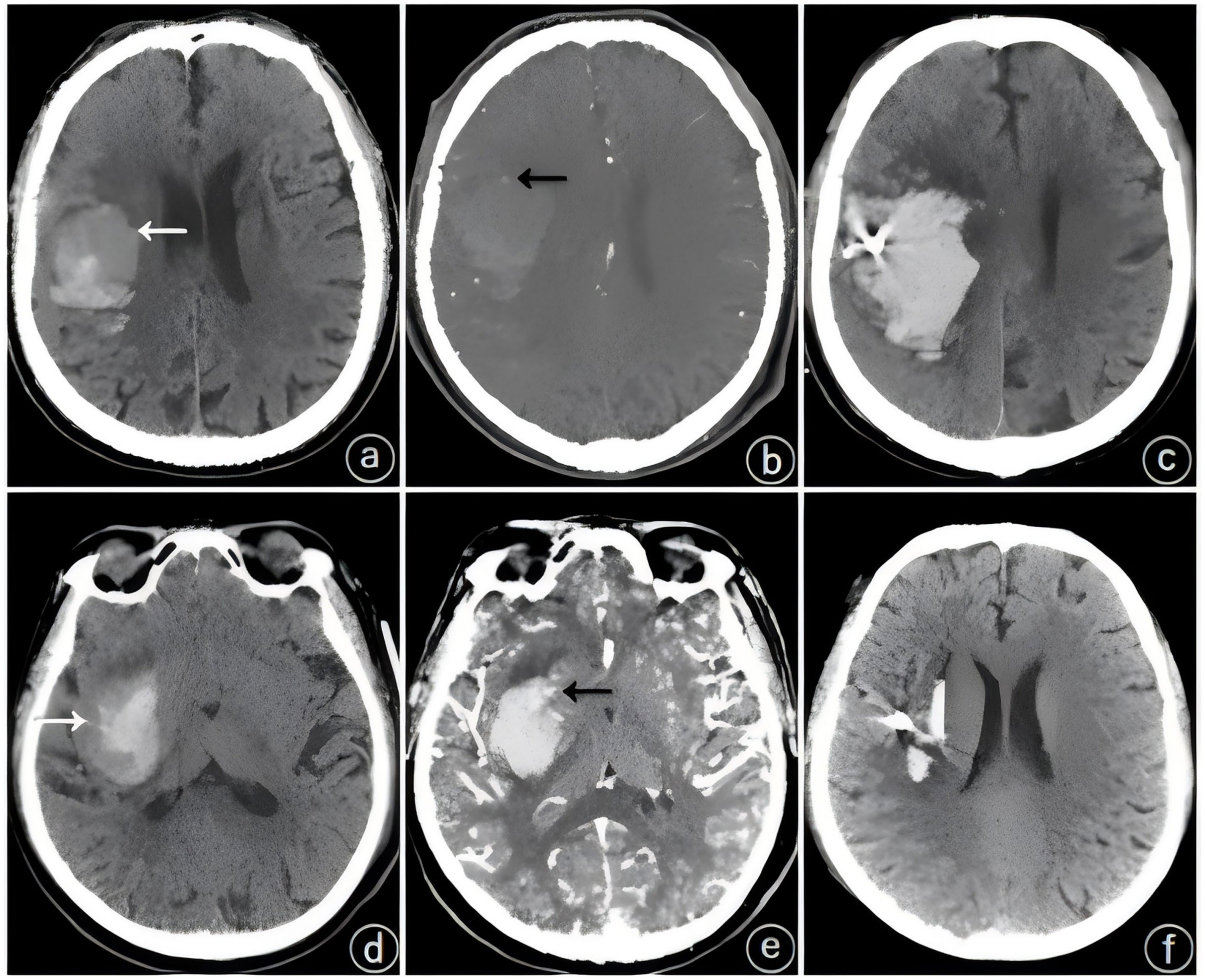
Typical cases of spot sign (black arrow) combined with blend sign (white arrow). **(a–c)** Patient: male, 77 years old, **(a)** initial hematoma of 36 mL; blend sign on computed tomography (CT); **(b)** spot sign on computed tomographic angiography (CTA) within 24 h; **(c)** HE on postoperative repeat computed tomography (CT); **(d)** Patient: male, 66 years old; initial hematoma of 27 mL; blend sign on computed tomography (CT); **(e)** spot sign on computed tomographic angiography (CTA) within 24 h; **(f)** no hematoma expansion (HE) on postoperative repeat computed tomography (CT).

The patients underwent initial cranial CT and CTA within 24 h of admission, and imaging signs, including the spot sign, the blend sign, and the spot sign combined with the blend sign, were recorded. Patients underwent CTA when they met the following criteria: (1) cranial CT-confirmed spontaneous ICH; (2) exclusion of hemorrhagic infarction, aneurysmal rupture, and tumor-related hemorrhage; and (3) surgical eligibility for hematoma evacuation with planned intervention. The blend sign was defined as a visible hematoma shadow on cranial CT consisting of two different density components with a difference of ≥18 HU between the CT values of the high-density and low-density shadows; furthermore, the relatively low-density areas were not encapsulated by the relatively high-density areas (8) (Figure 1). The spot sign was defined as a visible hematoma with one or more dense shadows with a maximum contrast diameter of ≥3 mm. At least one focus of contrast extravasation was observed on ≥2 planes within the hematoma, which appeared in the arterial phase and persisted in the venous phase (Figure 2) (16). The presence of both the blend sign and the spot sign was defined as a combined sign (Figure 3). Two experienced physicians independently interpreted the imaging signs; in cases of inconsistency in the assessment, the decision was made through a joint discussion between the two physicians.

### 2.3 Prognostic assessment

The patients were followed up 90 days after the onset of ICH, and their mRS scores were assessed during this period. An mRS score of ≤3 indicated a good outcome, whereas a score of >3 was considered a poor outcome. The 195 patients with ICH were categorized into poor or good outcome groups.

### 2.4 Stereotactic minimally invasive surgical methods and postoperative management

After local anesthesia, the stereotactic frame was affixed, CT localization was performed, the puncture level was selected, the right-angle coordinate system was established, the target point of hematoma puncture was confirmed, and the *X*-, *Y*-, and *Z*-axis coordinate values of each target point were calculated, as were the length of the puncture needle and the depth of the needle entry. The puncture needle penetrated the skull and meninges, reached the target point, and slowly aspirated the hematoma. After the operation, we evaluated whether tube drainage was smooth and whether there was active bleeding; furthermore, we promptly reviewed the head CT image to observe residual intracranial hematoma (13, 14).

### 2.5 Statistical methods

Statistical analysis was performed using SPSS 25.0 (IBM Corp., Armonk, NY, United States). Data that conformed to a normal distribution are expressed as the means ± SDs, and an independent samples *t*-test was used for comparisons between groups. Non-normally distributed continuous variables were summarized as median with interquartile range (IQR), with intergroup comparisons assessed using the Mann–Whitney *U*-test. Count data are expressed as the number of cases and percentages, and the *χ*^2^ test or Fisher’s exact probability method was used for comparisons between groups. Data from the three groups were compared using a one-way ANOVA. For the identification of outcome-influencing factors, variables meeting the *p-*value of <0.05 threshold in univariate screening were modeled via logistic regression. The predictive value of each imaging characteristic was analyzed via the receiver operating characteristic (ROC) curves.

## 3 Results

### 3.1 Baseline characteristics

**Figure 4:**
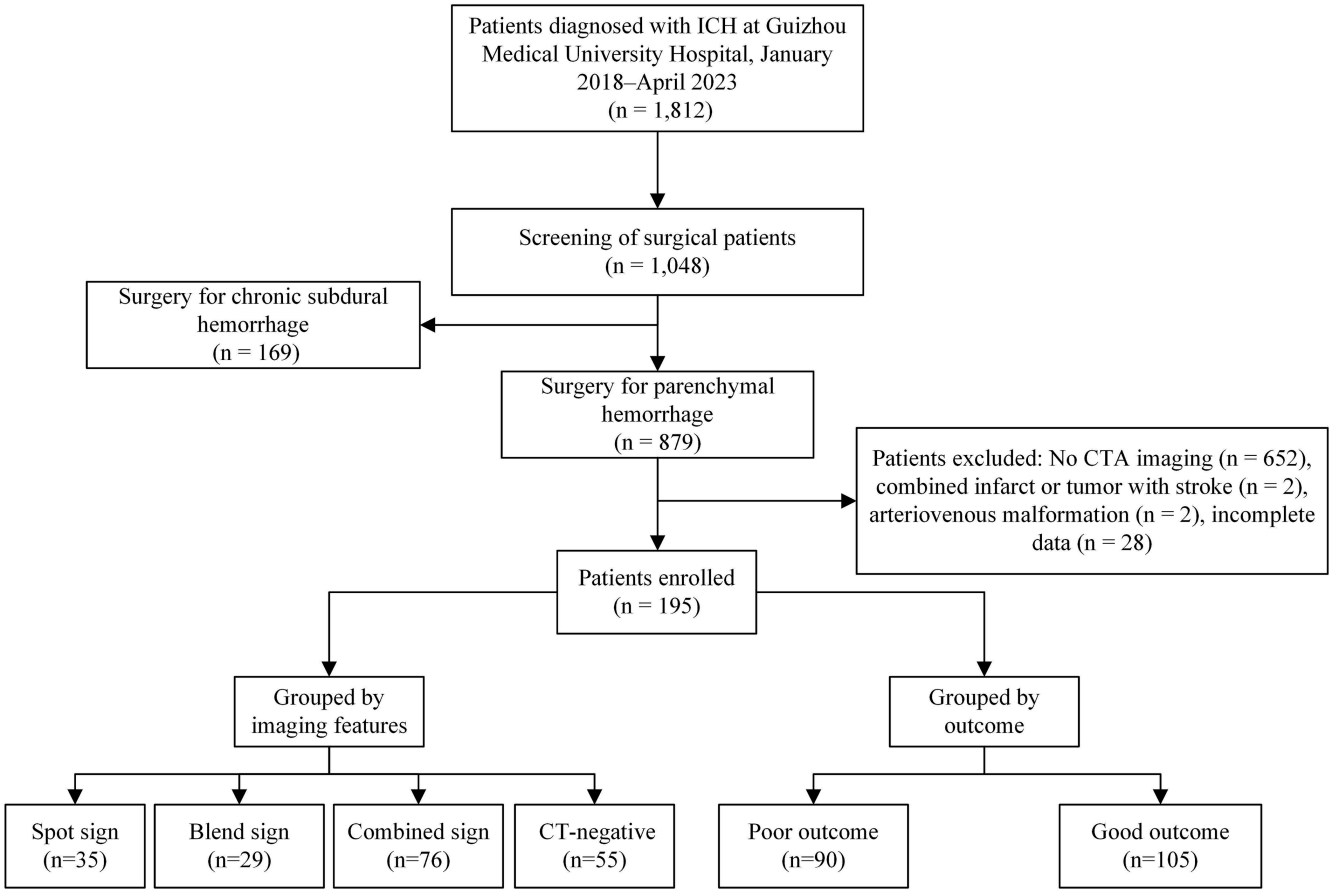
Flowchart of enrollment in this study.

Among the 879 patients with ICH who underwent sMIS, 195 were eligible for enrollment according to the inclusion and exclusion criteria. The patients were categorized into the poor outcome (90 patients) and good outcome (90 patients) groups according to their 90-day post-disease prognosis (Figure 4).

### 3.2 Comparison of baseline data across the four groups

According to the CT and CTA imaging characteristics, the clinical baseline data of the single spot sign group (*n* = 35), single blend sign group (*n* = 29), the combined group (*n* = 76), and the CT-negative group (*n* = 55) were compared, revealing no differences in age, smoking history, sex, systolic blood pressure, drinking history, baseline hematoma volume, NIHSS score on admission, or whether or not a ruptured ventricle occurred (*p* > 0.05). Patients in the combined sign group exhibited significantly higher admission systolic blood pressure and diastolic blood pressure compared to those in the single spot sign group, the single blend sign group, and the CT-negative group. The highest rate of HE was observed in patients with the spot sign combined with the blend sign, which was significantly greater than that in patients with the single blend sign, the single spot sign, or the CT-negative group (*p* < 0.05) (Table 1).

**Table 1:** Comparison of the general baseline data across the four groups.

Characteristics	Single spot sign (*n* = 35)	Single blend sign (*n* = 29)	Combined sign (*n* = 76)	CT-negative (*n* = 55)	T/F	*p-*value
Mean age, yr.	59.89 ± 10.94	59.54 ± 9.56	56.69 ± 13.29	58.06 ± 12.09	0.617^a^	0.605
Sex, male (%)	20 (57.1)	20 (71.4)	33 (64.7)	26 (47.2)	6.609^b^	0.085
Smoking (%)	18 (51.4)	14 (50.0)	29 (38.2)	15 (27.3)	6.525^b^	0.089
Alcohol consumption (%)	17 (48.6)	12 (42.9)	31 (45.3)	19 (34.5)	3.778^b^	0.286
Hypertension (%)	18 (51.4)	15 (53.6)	50 (66.7)	35 (63.6)	5.682^b^	0.460
Diabetes mellitus (%)	3 (8.6)	2 (7.1)	3 (4.0)	7 (12.7)	3.529^b^	0.317
Systolic pressure	156.5 ± 37.4	157.5 ± 22.1	179.1 ± 25.6	162 ± 29.0	2.885^a^	0.037
Diastolic pressure	98.21 ± 18.0	92.82 ± 11.8	101.9 ± 16.1	96.5 ± 18.3	5.179^a^	0.002
Baseline intracerebral hemorrhage volume	32.1 ± 17.7	32.6 ± 14.8	36.5 ± 14.9	31.4 ± 16.0	2.289^a^	0.080
Ruptured into the ventricle (%)	11 (31.4)	5 (17.9)	19 (25.3)	23 (41.8)	7.076^b^	0.070
HE (%)	13 (37.1)	11 (37.9)	35 (46.1)	6 (10.9)	11.574^b^	0.009
NIHSS on admission [*M*(*P*_25_, *P*_75_), score]	12 (9, 15)	14 (8, 18)	13 (9.5, 17)	16 (5, 22)	7.179^c^	0.066

^a^ is the *t*-value; ^b^ is the *χ*^2^ value; and ^c^ is the *Z*-value.

### 3.3 Comparison of clinical data between the outcome groups

There was no significant difference in age, sex, history of diabetes mellitus, history of hypertension, history of smoking, history of alcohol consumption, volume of hematoma on admission, duration of drain retention, intracranial pressure before hematoma removal, intracranial pressure after hematoma removal, admission glucose, serum calcium, serum sodium, cystatin, or hyperhomocysteinemia between the poor and good outcome groups. Nevertheless, systolic blood pressure, diastolic blood pressure, NIHSS score, and proportion of HEs were greater in the poor outcome group than in the good outcome group, and the GCS score was significantly lower in the poor outcome group than in the good outcome group. A comparative analysis of hematoma topography between the groups revealed that, in the poor outcome cohort (*n* = 90), hematomas predominantly involved the basal ganglia (65, 72.2%), followed by lobar regions (20, 22.2%), with fewer cases in the thalamus (2, 2.2%), cerebellum (2, 2.2%), and brainstem (1, 1.1%). The favorable outcome group (*n* = 105) demonstrated higher proportions of lobar (47, 44.8%) and cerebellar (7, 6.7%) hemorrhages, whereas basal ganglia involvement remained frequent (50, 47.6%) (*p* < 0.01). Radiographically, the mean residual hematoma volume decreased from 34.4 mL on baseline CT scans to 6.28 mL postoperatively. In terms of imaging characteristics, there was no significant difference in the proportions of patients with the blend sign and those with the spot sign between the two groups (*p* > 0.05). In the poor outcome group, the proportion of patients with the two combined signs was significantly greater than that in the good outcome group (*p* < 0.001). Moreover, the proportions of patients with postoperative pulmonary infection and gastrointestinal bleeding in the poor outcome group were significantly greater than those in the good outcome group (Table 2).

**Table 2:** Comparison of the clinical data of patients in the poor and good outcome groups.

Characteristics	Poor outcome (*n* = 90)	Good outcome (*n* = 105)	*T*/*F*	*p*
Age [*M*(*P*_25_, *P*_75_), years]	56 (48, 66)	57 (49, 66)	−0.211^c^	0.833
Sex, male (%)	60 (66.7)	63 (60.0)	0.925^b^	0.336
Diabetes mellitus (%)	5 (5.6)	10 (9.5)	1.075^b^	0.300
Hypertension (%)	58 (64.4)	61 (58.1)	2.188^b^	0.335
Smoking (%)	33 (36.7)	43 (41.0)	0.374^b^	0.541
Alcohol consumption (%)	38 (42.2)	46 (43.8)	0.050^b^	0.823
Systolic pressure [*M*(*P*_25_, *P*_75_), mmHg]	170.0 (155.0, 189.0)	157.0 (138.0, 183.0)	−3.108^c^	0.002
Diastolic pressure (*x* ± *s*, mmHg)	105.3 ± 20.0	97.6 ± 17.8	2.846^a^	0.005
Hyperhomocysteinemia (%)	29 (32.2)	28 (26.7)	2.749^b^	0.253
Admission glucose (mmol/L)	6.9 (5.9, 7.9)	6.9 (6.1, 8.1)	−0.691^c^	0.490
Cystatin C (mg/L)	0.99 (0.81, 1.27)	0.97 (0.83, 1.12)	−0.615^c^	0.539
Serum calcium (mmol/L)	2.24 (2.16, 2.32)	2.26 (2.20, 2.34)	−1.685^c^	0.092
Serum sodium (mmol/L)	140.80 (138.74, 142.80)	140.46 (137.90, 142.35)	−0.928^c^	0.354
Dyslipidemia (%)	30 (33.3)	27 (25.7)	2.112^b^	0.348
GCS on admission [*M*(*P*_25_, *P*_75_), score]	12 (10, 14)	13 (12, 14)	−3.073^c^	0.002
NIHSS score on admission [*M*(*P*_25_, P_75_), score]	15 (12, 21)	10 (4, 16)	−5.288^c^	0.000
Baseline ICH volume (*x* ± *s*, ml)	36.7 ± 17.2	32.5 ± 16.1	1.761^a^	0.080
Drainage tube retention time (d)	4 (3, 6)	4 (3, 5)	−1.308^c^	0.191
Preoperative ICP [*M*(*P*_25_, *P*_75_), mmHg]	24.0 (16.0, 29.0)	24.0 (17.0, 28.0)	−0.335^c^	0.738
Postoperative ICP [*M*(*P*_25_, *P*_75_), mmHg]	12.0 (8.0, 15.0)	12.0 (7.0, 15.0)	−0.421^c^	0.674
Admission to ICU (%)	7 (7.8)	7 (6.7)	1.276^b^	0.528
Ruptured into ventricle (%)	32 (35.6)	27 (25.7)	2.224^b^	0.136
Hematoma expansion (%)	38 (42.2)	21 (20.0)	11.341^b^	0.001
Postoperative lung infection (%)	74 (82.2)	72 (68.8)	4.800^b^	0.028
Postoperative heart failure (%)	7 (7.8)	7 (2.9)	2.421^b^	0.120
Postoperative gastrointestinal bleeding (%)	33 (36.7)	17 (16.2)	11.283^b^	0.004
Single blend sign (%)	9 (10.0)	20 (19.0)	3.133^b^	0.077
Single spot sign (%)	11 (12.1)	24 (22.9)	3.722^b^	0.054
Spot sign combined with blend sign (%)	55 (61.1)	21 (20.0)	34.437^b^	0.000
Hematoma location (%)			17.453^b^	0.004
Basal ganglia	65 (72.2)	50 (47.6)		
Lobar	20 (22.2)	47 (44.8)		
Thalamus	2 (2.2)	0 (0)		
Cerebellum	2 (2.2)	7 (6.7)		
Brainstem	1 (1.1)	1 (1)		

^a^ is the *t*-value; ^b^ is the *χ*^2^ value; and ^c^ is the *Z*-value.

### 3.4 Risk factors for poor outcomes

Multivariate binary logistic regression analysis was performed, considering the following variables: diastolic blood pressure at admission, systolic blood pressure at admission, spot sign combined with blend sign, HE, postoperative lung infection, and postoperative gastrointestinal bleeding. The results revealed that the spot sign combined with the blend sign (OR = 5.244, 95% CI: 2.606–10.55, *p* < 0.001) and HE (OR = 2.063, 95% CI: 1.003–4.245, *p* = 0.049) were associated with poor outcomes (*p* < 0.001), suggesting that the spot sign combined with the blend sign and HE were correlates of poor outcomes (Table 3).

**Table 3:** Binary logistic regression analysis.

Factors	*B*	*SE*	Wald *χ*^2^	OR	95%CI	*p*
Systolic pressure	0.012	0.009	1.692	1.012	0.994–1.03	0.193
Diastolic pressure	−0.002	0.014	0.028	0.867	0.971–1.025	0.867
Spot sign combined with blend sign	1.657	0.357	21.585	5.244	2.606–10.55	0.000
Hematoma expansion	0.724	0.368	3.871	2.063	1.003–4.245	0.049
Postoperative lung infection	0.761	0.416	3.356	2.141	0.948–4.834	0.067
Postoperative gastrointestinal bleeding	0.665	0.381	3.042	1.944	0.921–4.104	0.081

### 3.5 ROC curve for outcome prediction in patients undergoing sMIS for ICH

**Figure 5:**
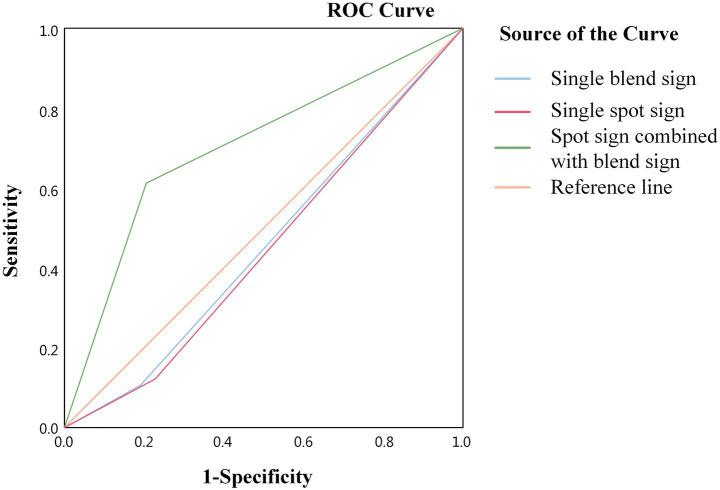
ROC curve analysis of the effect of combined signs on the outcome of patients with ICH.

The ROC curves were plotted, and the sensitivity, specificity, and Youden index of each imaging feature in predicting poor outcomes were calculated. The results revealed that the sensitivity and specificity of a single blend sign affecting the outcome of patients with ICH who have undergone sMIS were 19 and 90%, respectively, and the Youden index was 0.09, with an area under the curve (AUC) of 0.545 (*p =* 0.277); those of the spot sign affecting the outcome were 22.9 and 87.8%, respectively, with a Youden index of 0.107, and the AUC was 0.553 (*p* = 0.201). The positive and negative predictive values of the combined sign were 72.4 and 71.8%, respectively. The sensitivity and specificity of the combined signs affecting outcome were 61.1 and 80%, respectively, with a Youden index of 0.411 and an AUC of 0.706 (*p* < 0.05) (Figure 5 and Table 4).

**Table 4:** Predictive value of different imaging signs for outcome.

Results	Blend sign	Spot sign	Combined signs
Sensitivity (%)	19.0	22.9	61.1
Specificity (%)	90.0	87.8	80.0
Youden index	0.09	0.107	0.411
Area under the curve	0.545	0.553	0.706

## 4 Discussion

This study aimed to predict the adverse outcomes of patients who have undergone sMIS for ICH by evaluating the combination of the spot and blend signs. Our results revealed that combined signs emerged as independent covariates for poor outcomes in patients with ICH who have undergone sMIS, whereas single spot or blend signs were not predictors of poor outcomes.

We investigated the presence and predictive capacity of the spot sign combined with the blend sign by incorporating other risk factors into a multivariate longitudinal modeling approach to assess short-term outcomes. This approach differs from that applied in most previous studies, which have typically focused on a single CT sign using multivariable logistic regression models to assess ICH expansion, functional outcomes, early recurrence, and early mortality. Previous studies have shown that CT markers such as hypodensities, island signs, and black hole signs can predict poor outcomes in ICH patients within 90 days, regardless of whether surgery was performed (17). In our previous study, the blend sign was identified as a predictor of postoperative HE in patients who had undergone sMIS for ICH. After further evaluation of the relationship between the blend sign and the prognosis of patients undergoing sMIS for ICH, it was found that the postoperative GCS and NIHSS scores improved in both the blend sign and non-blend sign groups, suggesting that the blend sign is not associated with poor prognosis in patients undergoing sMIS (13, 14). In another study focusing on patients undergoing craniotomy, the significant factor affecting their prognosis was irreversible brain damage from the hematoma volume, and the blend sign did not predict patient prognosis (18). Compared to the osmotic sign, the spot sign is less sensitive and specific in predicting HE, and the osmotic sign predicts poor prognosis in patients, whereas the spot sign does not (19). Another study investigated the relationships between the blend sign, spot sign, hematoma volume, and GCS score on HE and poor prognosis after ICH and revealed that a GCS score of ≤8 and postoperative HE were significant predictors of poor prognosis and that the spot sign or blend sign was not a risk factor for postoperative HE or poor prognosis (20). Recent research has shown that spot signs are frequently observed in patients with ICH and that elevated spot sign scores are correlated with subsequent HE and prolonged hospital stays; however, they are not associated with adverse outcomes (21). The conclusions of this study are consistent with those of other studies that revealed that a single blend sign or spot sign did not predict prognosis in patients with ICH who have undergone sMIS.

The presence of either the spot sign or the blend sign is associated with increased mortality, HE, and poorer functional outcomes in patients with ICH, underscoring the predictive significance of these signs. However, these studies did not differentiate between surgical and non-surgical patients, thus masking the prognostic significance of blend or spot signs in patients undergoing sMIS (22–24). Another study explored the relationship between the blend sign and poor 90-day prognosis in 238 patients with ICH, including 16.8% of patients with the blend sign, accounting for 75% of patients with poor prognosis, and revealed that age, intraventricular hemorrhage, baseline hematoma volume, and the blend sign independently predict poor 90-day function (25). Another study explored the value of the CTA spot sign for postoperative HE and poor 90-day prognosis in patients with ICH treated with endoscopic hematoma debridement. The incidence of postoperative HE was greater in the spot sign-positive group than in the spot sign-negative group (25.0% vs. 6.8%). This study revealed that the spot sign is an independent predictor of poor 90-day prognosis in patients who underwent surgery (26). These two studies were inconsistent with the present study, where a single spot sign or blend sign predicted poor prognosis in ICH patients, which may be related to the bias introduced by the treatment modality and grouping criteria.

The findings of this study indicated that spot signs combined with blend signs retained independent prognostic significance for poor outcomes in patients with ICH who have undergone sMIS. Researchers have investigated the relationship between spot and blend signs in non-surgical patients, revealing that these signs are highly correlated. The spot sign and blend sign independently predict early neurological deterioration and poor prognosis in patients with ICH (27, 28). To increase the predictive efficacy of the blend sign and spot sign for poor prognoses among patients who have undergone sMIS for ICH, in this study, we defined the combined sign and explored the value of the spot sign and blend sign combined in predicting poor prognoses among these patients. Our results revealed that the combination of the spot sign and blend sign extends to functional outcomes, serving as a potential prognostic indicator for patients who have undergone sMIS.

This study has several limitations. First, despite efforts to increase the sample size, it remained relatively small, and the study was conducted at a single stroke center, underscoring the necessity for additional multicenter studies to corroborate our findings. Second, to ensure uniformity in the pathogenesis of our patients’ conditions, we excluded individuals with ICH resulting from secondary cerebral hemorrhage, hemorrhagic conversion of ischemic stroke, or structural lesions, as well as those who were treated conservatively with medication. Consequently, the conclusions drawn from our study do not pertain to these specific patient groups. Further investigations are warranted to explore the CT or CTA sign characteristics among patients with diverse types of ICH. Our findings are based on a retrospective, chart-based analysis. Since CTA is not routinely performed for intraparenchymal hemorrhage, 652 patients with ICH were excluded, potentially introducing selection bias and limiting the generalizability of our study. Finally, in ICH, hematoma topography is an independent prognostic determinant. Our analysis suggests a potential effect modification between hemorrhage location and primary study outcomes.

## 5 Conclusion

The spot sign combined with the blend sign and hematoma topography has a specific predictive value for poor outcomes in patients with ICH who have undergone sMIS.

## Data Availability

The raw data supporting the conclusions of this article will be made available by the authors, without undue reservation.
